# Complete *APTX* deletion in a patient with ataxia with oculomotor apraxia type 1

**DOI:** 10.1186/s12881-015-0213-y

**Published:** 2015-08-19

**Authors:** Rick van Minkelen, Miriam Guitart, Conxita Escofet, Grace Yoon, Peter Elfferich, Galhana M. Bolman, Robert van der Helm, Raoul van de Graaf, Ans M.W. van den Ouweland

**Affiliations:** Department of Clinical Genetics, Erasmus Medical Center, P.O. Box 2040, Rotterdam, 3000 CA The Netherlands; Genetic Laboratory, UDIAT-Centre Diagnòstic, Neuropediatrics Unity, Corporació Sanitària Universitària Parc Taulí, Sabadell, Spain; Division of Clinical and Metabolic Genetics, Department of Pediatrics, The Hospital for Sick Children and University of Toronto, Toronto, Canada

**Keywords:** *APTX*, Ataxia with oculomotor apraxia type 1, Deletion, Homozygous, MLPA, SNP array analysis, Breakpoint mapping, Genetic testing

## Abstract

**Background:**

Ataxia with oculomotor apraxia type 1 is an autosomal-recessive neurodegenerative disorder characterized by a childhood onset of slowly progressive cerebellar ataxia, followed by oculomotor apraxia and a severe primary motor peripheral axonal motor neuropathy. Ataxia with oculomotor apraxia type 1 is caused by bi-allelic mutations in *APTX* (chromosome 9p21.1).

**Case presentation:**

Our patient has a clinical presentation that is typical for ataxia with oculomotor apraxia type 1 with no particularly severe phenotype. Multiplex Ligation-dependent Probe Amplification analysis resulted in the identification of a homozygous deletion of all coding *APTX* exons (3 to 9). SNP array analysis using the Illumina Infinium CytoSNP-850 K microarray indicated that the deletion was about 62 kb. Based on the SNP array results, the breakpoints were found using direct sequence analysis: c.-5 + 1225_*44991del67512, p.0?. Both parents were heterozygous for the deletion. Homozygous complete *APTX* deletions have been described in literature for two other patients. We obtained a sample from one of these two patients and characterized the deletion (156 kb) as c.-23729_*115366del155489, p.0?, including the non-coding exons 1A and 2 of *APTX*. The more severe phenotype reported for this patient is not observed in our patient. It remains unclear whether the larger size of the deletion (156 kb vs 62 kb) plays a role in the phenotype (no extra genes are deleted).

**Conclusion:**

Here we described an ataxia with oculomotor apraxia type 1 patient who has a homozygous deletion of the complete coding region of *APTX*. In contrast to the patient with the large deletion, our patient does not have a severe phenotype. More patients with deletions of *APTX* are required to investigate a genotype-phenotype effect.

**Electronic supplementary material:**

The online version of this article (doi:10.1186/s12881-015-0213-y) contains supplementary material, which is available to authorized users.

## Background

Ataxia with oculomotor apraxia type 1 (AOA1) is an autosomal-recessive neurodegenerative disorder mainly characterized by a childhood onset of slowly progressive cerebellar ataxia, oculomotor apraxia, dysarthria, limb dysmetria, motor and sensory axonal neuropathy [1–3]. Clinical symptoms can also include dystonia, chorea, optic atrophy and cognitive impairment. AOA1 is caused by homozygosity or compound heterozygosity for mutations in *APTX*, the gene that encodes the protein Aprataxin [4, 5]. Aprataxin is a member of the histidine triad (HIT) superfamily and plays a role in DNA-single-strand break repair [6–12]. The pathological mechanism leading to the neurodegenerative phenotype, as observed for AOA1 patients, is still unknown.

In the current report we describe the extensive clinical and molecular genetic testing of an AOA1 patient who is homozygous for a complete *APTX* deletion rather than compound heterozygous for point mutations in *APTX* as is normally found in AOA1 patients. We also introduce the Leiden Open (source) Variation Database for *APTX* mutations.

## Case presentation

### Case report

Our index patient is the first child of healthy non-consanguineous Moroccan parents. He has two healthy brothers and parents refer no family history of interest. He was born after a normal gestation and both delivery and neonatal period were unremarkable. He started walking around 12 months and his parents considered his language development normal during the first years. At age 3 years, his parents started to notice abnormal ocular movements, walking disorder with frequent falls and language difficulties. He was clinically diagnosed with cerebellar ataxia. Cranial Computed Tomography and cranial Magnetic Resonance imaging (MRI) scans showed cerebellar atrophy (for MRI scan see Additional file 1). At age 6 years the child was referred to the Neuropaediatrics unit of the Sabadell university hospital in Spain. Neurological exam showed oculomotor apraxia, ataxic gait and dysmetria. Furthermore, he also presented distal limb dystonia, bilateral spontaneous Babinski and rotulian and achillean hyporeflexia. Blood levels of albumin, cholesterol, immunoglobulins, alpha-fetoprotein and carcinoembryonic antigen were all normal. Vitamin E levels at age 7 years, however, were low (repeated measurements: 1.3 μg/ml and 0.4 μg/ml; normal range 3–15 μg/ml). Because of the low vitamin E levels, molecular testing of the gene involved in vitamin E deficiency, *TTPA*, was performed [13]. No abnormalities were observed. Vitamin E levels returned to normal (11.7 μg/ml) after oral vitamin E treatment. Results of the Brainstem Auditory Evoked Response (BAER) test and the ophthalmologic and cardiological assessments were all normal. Electromyography (EMG) at age 7 years showed a discrete reduction of the sural sensory nerve action potential (SNAP) amplitude. At age 9 years the EMG results were in agreement with sensitive axonal polyneuropathy. No abnormalities were found in the gene involved in Ataxia-telangiectasia, *ATM* [14]. Finally, molecular testing of *APTX* was performed.

### APTX sequencing and MLPA

All seven coding exons (exons 3 to 9B) and exon/intron boundaries of *APTX* (NM_175073.2, isoform a, GRCh build 37 (UCSC hg19, February 2009)) were screened using direct sequence analysis (primers available upon request). Although we have previously used this setup to test for mutations in *APTX* (n = 158 index patients tested, 17 different *APTX* variants found (for 9 novel *APTX* variants see Additional file 2), also see http://www.lovd.nl/aptx, in total 45 *APTX* sequence variants), we were unable to amplify all exons, raising the possibility that *APTX* was not present on both alleles (homozygous deletion) of our patient. Therefore, the SALSA Multiplex Ligation-dependent Probe Amplification (MLPA) kit P316 (MRC Holland, Amsterdam, The Netherlands) was used to detect large deletions in *APTX*. MLPA analysis was performed according to manufacturer’s instructions. MLPA products were run on an ABI 3730XL automated sequencer (Applied Biosystems, Foster City, CA, USA) and data was analyzed using Genemarker software version 2.4 (Softgenetics, State College, PA, USA). MLPA analysis resulted in the identification of a deletion of exons 3 to 9. The non-coding exons 1 and 2 were not deleted. The peak ratio was clearly zero instead of 0.5, indicating that the deletion was homozygous and not heterozygous. The MLPA results were in agreement with the sequence analysis findings.

### SNP array analysis

**Fig. 1:**
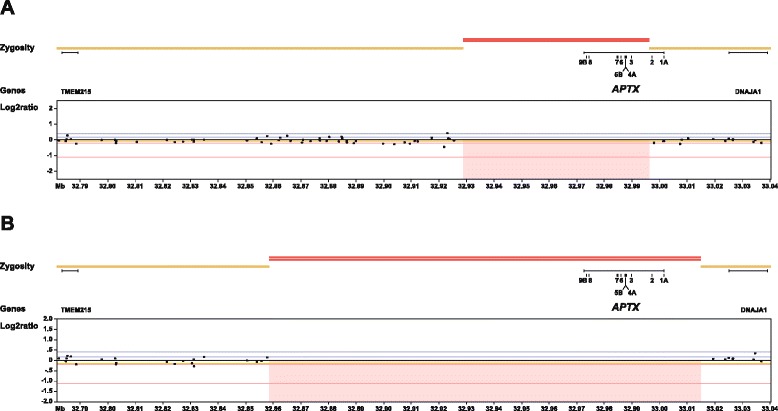
Results SNP array analysis. Indicated in the figure are the zygosity (heterozygous: yellow bars; homozygous: red bars) and Log2Ratio (reds square: deleted region based on Log2Ratio) tracks, the locations of *APTX* and surrounding genes on chromosome 9p21.1 (NCBI 37; UCSC hg19), and the exon numbering of *APTX* for our index patient (**a**) and the reference sample of Dr. Yoon et al. (**b**)

SNP array analysis was performed to confirm the MLPA finding and to further characterize the deletion. The Illumina Infinium CytoSNP-850 K microarray was used in combination with the Illumina platform and the Nexus Copy Number 7.0 software (BioDiscovery, El Segundo, CA, USA) according to standard protocols of the manufacturer. A deletion of 62 kb between positions 32.932.194 and 32.994.500 on chromosome chr9p21.1 (NCBI 37; UCSC hg19) was found (see Fig. 1a). Both the Log2ratio and the b-allele frequency (not shown in Fig. 1) indicate that the deletion is present in homozygous form. The deletion is in agreement with the MLPA results.

### Breakpoint mapping

Breakpoint mapping of the deletion was performed to characterize the exact breakpoints of the deletion. Based on the SNP array results, the breakpoints were found using direct sequence analysis. The exact nomenclature of the *APTX* deletion is c.-5 + 1225_*44991del67512, p.0?. A breakpoint specific PCR was developed (primers available upon request, for more details see reference [15]), confirming the homozygous occurrence of the deletion in the index patient. Both parents were heterozygous for the c.-5 + 1225_*44991del67512, p.0? deletion and were therefore identified as carriers of AOA1.

## Discussion

Most of the AOA1 patients are homozygous or compound heterozygous for a pathogenic point mutation in *APTX*. In the present report we describe a AOA1 patient who has a homozygous deletion of the complete coding region of *APTX*. Homozygous *APTX* deletions have been previously reported in a Tunisian family and a Pakistani patient [16, 17]. These deletions were found using southern blotting, after sequence fragments of all exons failed to amplify. Confirmation and characterization with more modern techniques like MLPA analysis and array analysis was not performed. Because their molecular analyses also lack breakpoint mapping we could not check whether our *APTX* deletion is the exactly the same deletion. We were however able to obtain a DNA sample of the patient of Yoon et al. SNP array analysis and breakpoint mapping, performed as described above, indicated a 156 kb *APTX* deletion,

c.-23729_*115366del155489, p.0? (APTX, exon 01A tm 09B), including the two non-coding *APTX* exons 1A and 2 (also see Fig. 1b).

The more severe phenotype reported for the patient of Yoon et al. [17], including fast deterioration and cognitive impairment, is not observed in our patient. It remains unclear whether the larger size of the deletion (156 kb vs 62 kb) plays a role in the phenotype. No extra genes are deleted, however, the two non-coding *APTX* exons 1 and 2B are deleted in the patient of Yoon et al. (also see Fig. 1). The function of these two exons is not studied. It is possible that this region contains regulatory elements that play in role in the transcription of (distance) genes, other than *APTX*, that might explain the more severe phenotype. Furthermore, a possible role of the different *APTX* transcripts in the AOA1 phenotype is not studied to date. Even though the predominant transcript in human tissues, NM_175073.2, contains both non-coding exons, several APTX transcripts exist that lack exons 1 and/or 2B [18]. Finally, a third option explaining the difference in phenotype between our patient and the patient of Yoon et al. is coincidence rather than a genetic feature. So far, genotype-phenotype correlations were only studied for a limited set of *APTX* point mutations [11, 12, 19]. More patients with deletions of *APTX* are required to investigate a genotype-phenotype effect. We were unfortunately unable to contact Amouri et al. to request a DNA sample [16]. Therefore, the size of the *APTX* deletion is unknown for their patient, neither do we know whether the two non-coding *APTX* exons are deleted in their patient. This is especially unsatisfactory because the typical AOA1 clinical presentation of our patient is similar to that reported for the patient of Amouri et al. [16].

To our knowledge, a database with *APTX* genetic variants was not available when writing this report. Because databases can be helpful in classifying variants we have launched a *APTX* database in LOVD (Leiden Open Variation Database) format [20]. All our mutations, including the total *APTX* deletion, and some well-known variants from literature were deposited in this database (accessible at http://www.lovd.nl/aptx). We will continue to update this database with new variants and encourage other *APTX* diagnostic labs to do the same.

## Conclusions

In conclusion, here we described the extensive clinical and genetic analysis of a AOA1 patient homozygous for a complete deletion of the coding exons of *APTX*. Our patient has a typical AOA1 clinical presentation without a severe phenotype. More patients with clearly characterized *APTX* deletions are required to investigate a genotype-phenotype effect.

## Consent

Written informed consent was obtained from the patient for publication of this Case report and any accompanying images. A copy of the written consent is available for review by the Editor of this journal

## Additional files

Additional file 1:
**MRI scan of index patient(white arrow).** (PDF 307 kb) Additional file 2:
**Novel APTX variants.** (PDF 65 kb)

## Abbreviations

AOA1: Ataxia with oculomotor apraxia type 1
BAER: Brainstem auditory evoked response
EMG: Electromyography
GTC: Genotyping console
MLPA: Multiplex ligation-dependent probe amplification
LOH: Loss of heterozygosity
LOVD: Leiden open variation database
SNAP: Sensory nerve action potential
QC: Quality control
