# Supplementary figures and images for: Complete APTX deletion in a patient with ataxia with oculomotor apraxia type 1
Source: BMC Med Genet. 2015 Aug 19;16:61. doi: 10.1186/s12881-015-0213-y (PMC4593195; doi:10.1186/s12881-015-0213-y)

Additional file 1

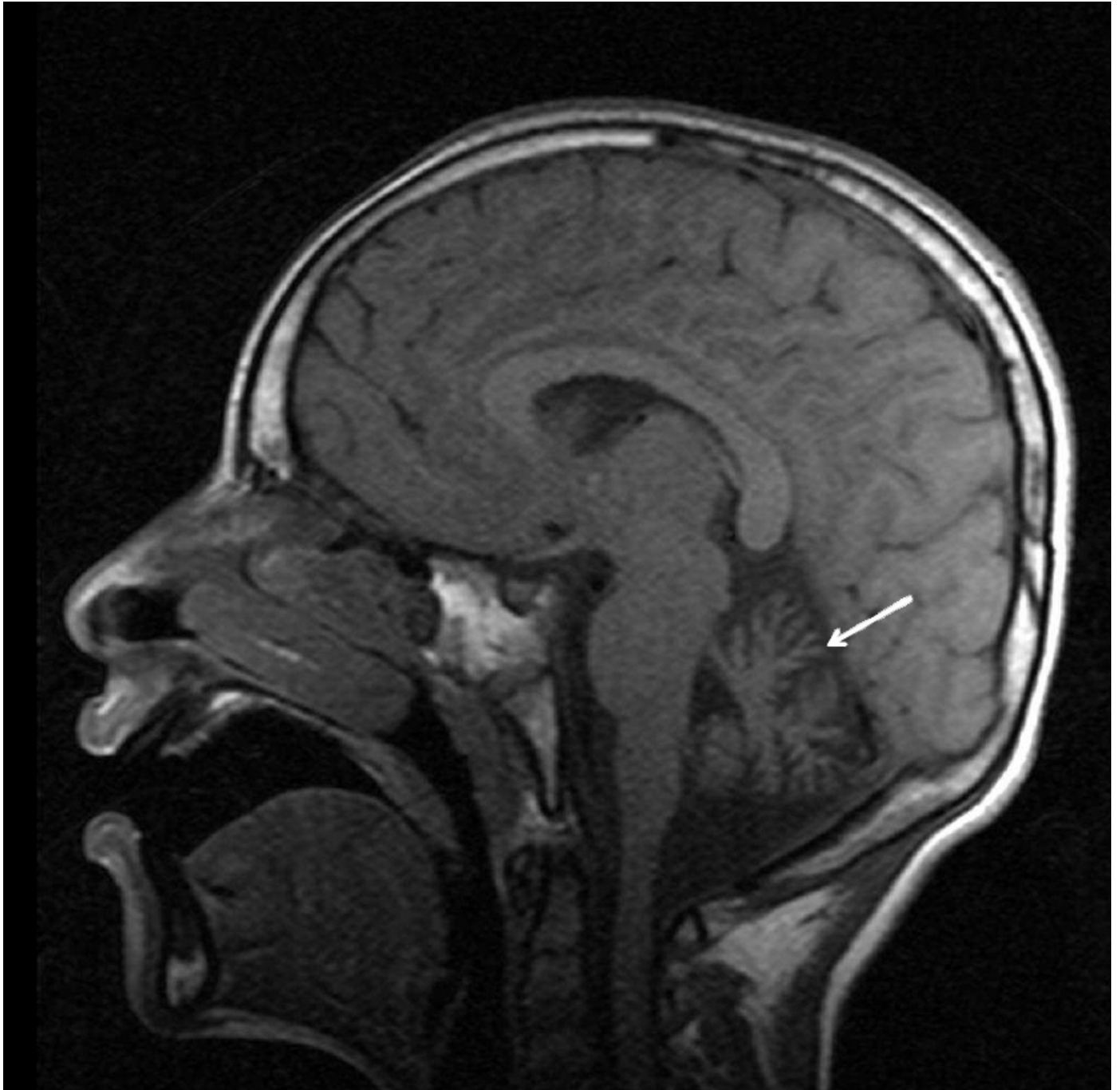

MRI scan of index patient showing atrophy (white arrow)

Supplement: Additional file 1: — MRI scan of index patient(white arrow). (PDF 307 kb) [file 12881_2015_213_MOESM1_ESM.pdf]
