# Supplementary material for: Complete APTX deletion in a patient with ataxia with oculomotor apraxia type 1
Source: BMC Med Genet. 2015 Aug 19;16:61. doi: 10.1186/s12881-015-0213-y (PMC4593195; doi:10.1186/s12881-015-0213-y)
Supplement: Additional file 2: — Novel APTX variants. (PDF 65 kb) [file 12881_2015_213_MOESM2_ESM.pdf]

Additional file 2

Novel APTX variants found in 158 index patients

ID	HGVS nucleotide change NM_175073.2	HGVS protein change	Exon	Pathogenicity*
27908	c.847A>G	p.(Asn283Asp)	8	3 (uncertain)
18647	c.940_956del17	p.(Lys314fs)	9B	5 (pathogenic)
23006	c.742T>A	p.(Leu248Met)	7	3 (uncertain)
4305	c.211G>T	p.(Val71Phe)	5B	3 (uncertain)
23263	c.971A>T	p.(Gln324Leu)	9B	1 (not pathogenic)
27320	c.559C>T	p.(Gln187*)	7	5 (pathogenic)
53383	c.318C>T	p.(=)	5B	2 (likely not pathogenic)
61291	c.203T>C	p.(Ile68Thr)	5B	3 (uncertain)
62385	c.18G>T	p.(Trp6Cys)	3	3 (uncertain)

* The software program Alamut version 2 (Interactive Biosoftware, Rouen, France) was used to help determine the pathogenicity of sequence variants as was previously described in detail in the supplemental data (pages 16-18) of Clin Genet. 2014 Apr;85(4):318-27.
